# Comparative Study of Self-Gripping Mesh vs. Polypropylene Mesh in Lichtenstein’s Open Inguinal Hernioplasty

**DOI:** 10.7759/cureus.43652

**Published:** 2023-08-17

**Authors:** Gautham Gunasekaran, Vamsi C Balaji, Surendran Paramsivam

**Affiliations:** 1 General Surgery, Sri Ramachandra Institute of Higher Education and Research, Chennai, IND

**Keywords:** self-gripping mesh, lichtenstein’s repair, open inguinal hernia repair, symptomatic hernia, hernia mesh, inguinal hernia surgery

## Abstract

Background

Inguinal hernia is one of the most common conditions in India, and history has many repair techniques recorded in it. Postoperative pain still remains a problem despite tension-free hernioplasty being accepted as the gold standard. Increased duration of surgery not only exposes the patient to unwanted increased chances of mesh infection but also reduces the surgeon's productivity if continued persistently. In this study, the main aim was to compare the fixation techniques of polypropylene mesh vs. self-gripping mesh in inguinal hernia surgery in terms of duration of surgery, postoperative pain, seroma, recurrence, foreign body sensation, and wound infections.

Methods

It is a prospective, comparative, and quantitative study conducted at Sri Ramachandra Institute of Higher Education and Research in the Department of General Surgery. Patients presenting with inguinal hernia to the OPD were included in the study. The sampling technique used in this study is simple, convenient sampling. As a result, the calculation of the margin of error and confidence levels may be difficult. Nevertheless, the sample accurately represents the population. Patients were divided into two groups: the study group (25), patients undergoing hernioplasty with self-gripping mesh, and the control group (25), patients undergoing hernioplasty with polypropylene mesh using conventional suturing. The duration of surgery, postoperative pain, seroma, recurrence, foreign body sensation, and wound infections were compared and analyzed between the two groups.

Results

In this study, the duration of surgery was less than one hour for three patients (12%) in the control group (polypropylene), compared to 13 (52%) patients in the study group (self-gripping), which is statistically significant. The early postoperative pain on POD 0 was greater than 4 (visual analogue score) in 8 (32%) patients in the control group and two (8%) patients in the study group. There were no significant differences in chronic pain, recurrence rate, seroma rate, or wound infection between the two groups.

Conclusions

In our study, we conclude that self-gripping mesh is superior to polypropylene mesh in surgery of inguinal hernia in terms of shorter duration of surgery. There is also reduced pain in the immediate postoperative period though not statistically significant. There is no significant difference in both the groups in terms of seroma formation, wound infection, foreign body sensation, and recurrence.

## Introduction

One of the most common diseases in society is inguinal hernia, and its repair modalities have been detailed extensively throughout history. The inguinal canal contains the round ligament in females and the spermatic cord in males. It has a tubular structure and runs inferomedially [1]. The processus vaginalis, an outpouching of the peritoneum, is attached to the testis and descends retroperitoneally into the scrotum. An inguinal hernia occurs when the processus vaginalis fails to obliterate [2]. The deep inguinal ring is a circular opening found in the fascia transversalis, one cm superior to the inguinal ligament and a cm lateral to the inferior epigastric artery, whereas the superficial inguinal ring is an inverted V-shaped opening in the external oblique aponeurosis, found lateral and superior to the pubic tubercle [3]. Low recurrence rates and morbidity in this surgery are attributed to advancements in perioperative anesthesia and operative techniques. Hence, avoiding chronic pain and improving the quality of life have become the most important considerations of hernia repair [4].

The gold standard for hernia repairs has been identified as tension-free repairs, but pain remains the main problem in the postoperative period. Hernias of the abdominal wall occur due to the displacement of intra-abdominal organs caused by the gaping of the muscles of the abdominal wall and the fascial layers, mesentery, or around the organs. Being one of the most common abdominal wall hernias, inguinal hernias are seen in both inguinal and femoral areas, and they are often categorized together. Groin hernias constitute 75% of abdominal wall hernias, being more common in 27% of men than in 3% of women [5]. Inguinal hernia repairs are performed in 90% of men and 10% of women. Its incidence in men has a bimodal distribution, with peaks just after birth and at over 40 years of age. According to the study by Abramson, the lifetime prevalence of inguinal hernias is 15% at the ages of 25-34 years and 47% at over 75 years of age [6].

Women undergo approximately 70% of total femoral hernia repairs. The most common variety of groin hernia is the indirect inguinal hernia, found in both men and women. Chronic pain occurs as a vital hindrance after hernia repair with mesh. Heavy-weight meshes made of polypropylene elicit inflammatory changes that are responsible for mesh shrinkage as it heals. Hence, lightweight meshes are preferred, and the extent of fixation is also limited. Recently, self-gripping meshes have been created to eliminate the need for additional fixation. The self-fixation mesh is made of monofilament polyester and polylactic acid grips (PLA), and it is indicated for repairing both inguinal and incisional hernias [7]. Despite the use of polypropylene mesh in inguinal hernia surgeries, self-gripping mesh is increasingly being preferred due to reduced postoperative pain and improved quality of life. As a result, patients return to normal routine activities quicker [8]. This study compares polypropylene mesh with conventional suturing to self-gripping mesh without suturing, using various criteria mentioned later in this paper.

## Materials and methods

This study was conducted at Sri Ramachandra Institute of Higher Education and Research in the Department of General Surgery from October 2020 to October 2022. Ethics committee approval (SRUPOC/2022/11733) was obtained before the start of the study. It was a prospective, comparative, and quantitative study. The sample size was calculated to be 50 patients, and they were divided into two groups through simple, convenient sampling and randomization. Group A (Study group) included 25 patients who had undergone open Lichtenstein hernioplasty with self-gripping mesh. In comparison, Group B (Control Group) included 25 patients who had undergone the same procedure with Polypropylene mesh. The technique of hernioplasty performed was Lichtenstein inguinal hernioplasty in both groups.

**Figure 1:**
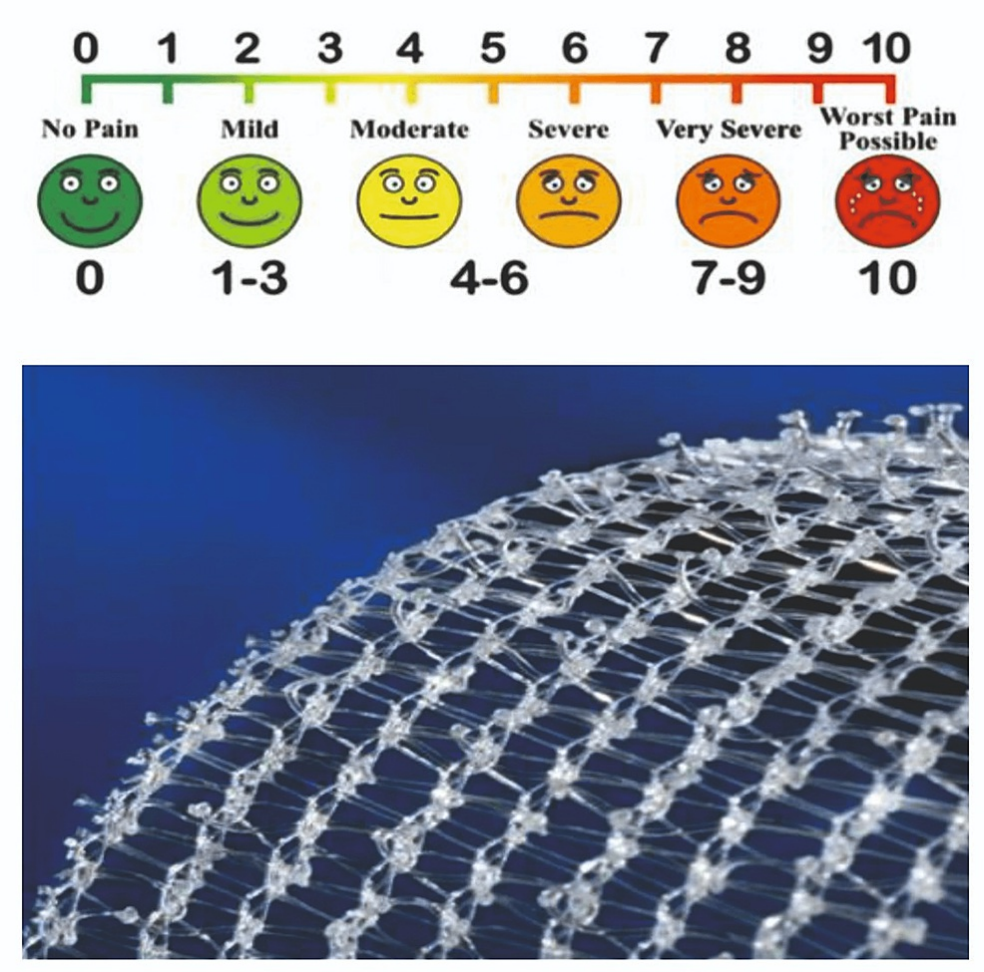
Illustration of the visual analogue score system and a self-gripping mesh.

The patients were followed for a period of three months, with the primary motive of assessing the duration of surgery. They were also followed up at six months and one year to check for recurrence. A shorter follow-up period was adopted in view of the early adoption of this technique, which will benefit the patients. The primary outcome was the duration of surgery, based on the number of hours, with the possible goal of achieving a shorter operation time. The secondary parameters assessed included postoperative pain using the visual analog score, seroma rate, wound infection, foreign body sensation, and recurrence of the hernia, all assessed through clinical judgment by the same surgeon, thereby improving the overall quality of life and enabling an early return to normal activities (Figure 1).

The data were collected from both groups, and the significance was statistically analyzed. The statistical method used here is the test of significance (Chi-square test, Fischer's exact test), along with correlation. Additionally, some descriptive analysis was done to strengthen the study.

Inclusion criteria

All patients with uncomplicated unilateral inguinal hernias, within the age group of 18-70 years, who were admitted to Sri Ramachandra Institute of Higher Education and Research in the Department of General Surgery and underwent Open Lichtenstein Hernioplasty during the study period, were included and followed up postoperatively as per protocol.

Exclusion criteria

Those patients with complicated Inguinal hernia [Obstruction or Strangulation], patients with recurrent inguinal hernia, patients undergoing the procedure under general anesthesia and the patients not willing to participate in the study were excluded. 

Materials used in the study

Patients with complicated inguinal hernia (such as obstruction or strangulation), those with recurrent inguinal hernia, patients undergoing the procedure under general anesthesia, and patients not willing to participate in the study were excluded. 

Operative steps

Under spinal anesthesia and strict aseptic conditions, patients in both groups underwent open Lichtenstein hernioplasty. After completing the basic dissection, delineating the sac, and reducing the contents, the study group that received the self-gripping mesh had it placed on the posterior wall without even a single suture, which might have been required for the medial border. In addition to this, a medial cover of two centimeters was also fulfilled. In contrast, the control group received conventional mesh fixation techniques. The mode of anesthesia given to all patients was spinal anesthesia. The study involved a single experienced surgeon who operated on both groups. After the surgery, patients were subsequently followed in the ward until they were discharged, and follow-up was done up to three and six months to assess the above parameters and one year for recurrence. Complications were treated accordingly, and all relevant investigations were conducted.

## Results

Age distribution

**Figure 2:**
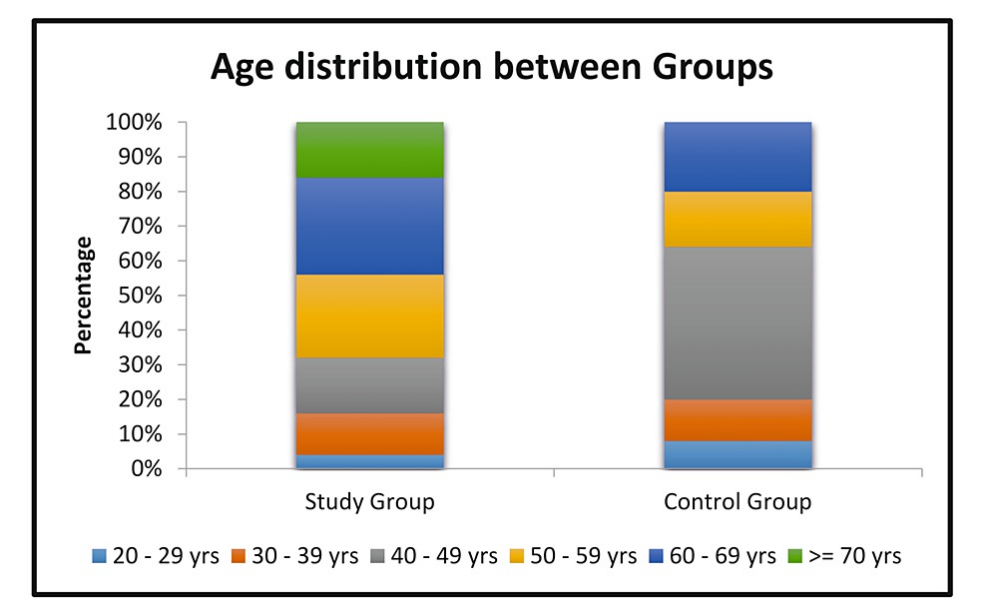
Age distribution between two groups

The total number of patients was 50, divided into two groups: the control group and the study group, each including 25 patients (Figure 2).

Mean age between two groups

**Figure 3:**
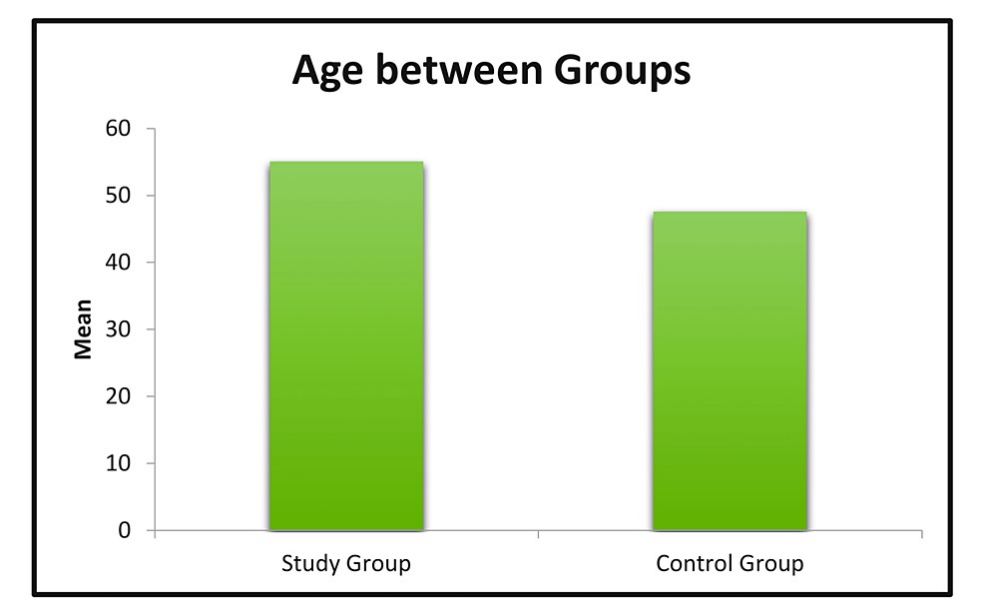
Mean age between two groups.

The mean age in the control group was 48 years, compared to the mean age of 55 years in the study group, a difference that was not statistically significant (Figure 3).

Side distribution between groups

**Figure 4:**
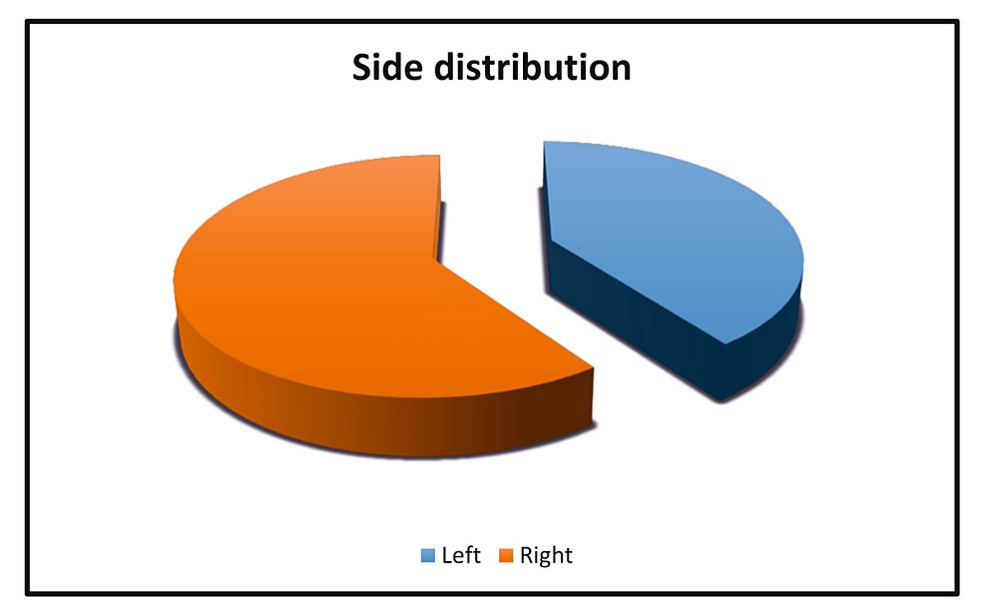
Side distribution between groups.

In the control group, there were 11 patients who presented with left inguinal hernia and 14 patients who presented with right inguinal hernia. A similar distribution was found in the study group, with nine patients presenting with left inguinal hernia and 16 patients with right inguinal hernia. This shows a higher prevalence on the right side, though the difference is insignificant (Table 1 and Figure 4).

**Table 1:** Side distribution between two groups.

Side distribution			Groups		Total	ꭓ2-value	P-value
			Study Group	Control Group			
Side	Left	Count	9	11	20	0.333	0.564 #
		%	36.0%	44.0%	40.0%		
	Right	Count	16	14	30		
		%	64.0%	56.0%	60.0%		
Total		Count	25	25	50		
		%	100.0%	100.0%	100.0%		
# No Statistical Significance at p > 0.05 level							

Pain score

Postoperative Pain on Post Operative Day (POD) Zero

Using the visual analogue scoring system, the pain score was evaluated in both groups. Values more than four were considered significant in this study.

In the study group, the pain score was less than four in 23 patients, as compared to 17 patients in the control group. However, only two patients had a pain score more than four in the study group, compared to eight in the control group. The correlation of pain on POD 0 between the two groups was not statistically significant (Table 2).

**Table 2:** Postoperative pain on POD 0. POD: Postoperative day.

Inguinal hernia			Groups		Total	ꭓ2-value	P-value
			Study Group	Control Group			
Pain post op day 0	< 4	Count	23	17	40	4.500	0.074 #
		%	92.0%	68.0%	80.0%		
	> 4	Count	2	8	10		
		%	8.0%	32.0%	20.0%		
Total		Count	25	25	50		
		%	100.0%	100.0%	100.0%		
# No Statistical Significance at p > 0.05 level							

Postoperative Pain on POD One

Control group:* *The study group had all its patients having a pain score under four. In contrast, there were four patients in the control group who had a pain score of more than four. The correlation of pain on POD 1 between the two groups was not statistically significant (Table 3).

**Table 3:** Postoperative pain on POD 1. POD: Postoperative day.

Inguinal hernia			Groups		Total	ꭓ2-value	P-value
			Study Group	Control Group			
Pain post op day 1	< 4	Count	25	21	46	4.348	0.110 #
		%	100.0%	84.0%	92.0%		
	> 4	Count	0	4	4		
		%	0.0%	16.0%	8.0%		
Total		Count	25	25	50		
		%	100.0%	100.0%	100.0%		
# No Statistical Significance at p > 0.05 level							

Postoperative Pain on POD Two

**Figure 5:**
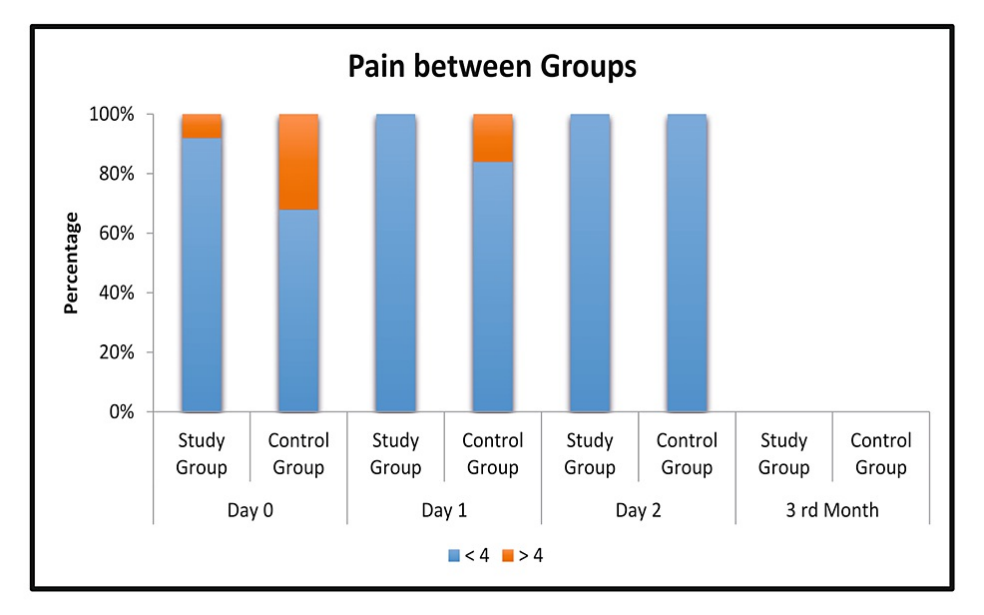
Pain score between groups.

The postoperative pain on POD 2 was less than 4 in both the groups. The correlation of pain on POD 2 between the two groups was not statistically significant (Figure 5).

Duration of surgery

The duration of surgery was less than one hour for 13 patients in the study group versus three patients in the control group. With a p-value of 0.005, which is statistically significant, this indicates that the duration of surgery is significantly less in the study group when compared to the control group. The table illustrates the chi-square test, and the figure shows the significance.

**Table 4:** Total duration of surgery. High statistical significance at p < 0.01 level.

Inguinal hernia			Groups		Total	ꭓ2-value	P-value
			Study Group	Control Group			
Surgery duration	< 1 hour	Count	13	3	16	9.191	0.005 **
		%	52.0%	12.0%	32.0%		
	> 1 hour	Count	12	22	34		
		%	48.0%	88.0%	68.0%		
Total		Count	25	25	50		
		%	100.0%	100.0%	100.0%		
** High statistical significance at p < 0.01 level							

**Figure 6:**
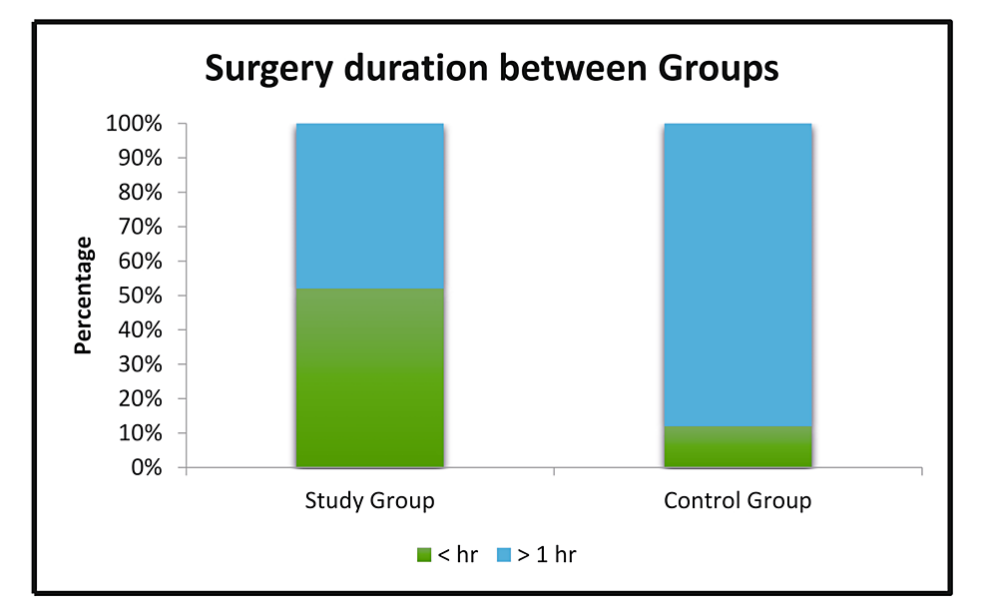
Significant difference in the duration of surgery between the two groups.

Seroma

None of the patients in the study group had seroma, compared to one patient in the control group who presented with seroma formation, which was treated accordingly. However, there was no significant difference in seroma rates between the study and the control group.

**Table 5:** Seroma rate between the two groups.

Inguinal hernia			Groups		Total	ꭓ2-value	P-value
			Study Group	Control Group			
Seroma	Absent	Count	25	24	49	1.020	1.000 #
		%	100.0%	96.0%	98.0%		
	Present	Count	0	1	1		
		%	0.0%	4.0%	2.0%		
Total		Count	25	25	50		
		%	100.0%	100.0%	100.0%		
# No statistical significance at p > 0.05 level							

 There were no wound infection, foreign body sensation, and recurrence at three months post-surgery in both the groups.

## Discussion

The inguinal hernia and its repair always pose a challenge and scope for improvement and advancement in the field of medicine. The mesh is placed on the posterior wall of the inguinal canal with a medial cover of approximately two centimeters medial to the pubic tubercle. Based on the review of the literature and the experiences, we believe that the fixation of mesh can be avoided, thereby preventing many untoward complications [9-11]. The postoperative pain on POD 0 and POD 1 was reduced in comparison with the control group, though it was statistically insignificant in the study. The lack of tension during mesh fixation and closure of the flap around the cord can reduce postoperative pain [12]. The tension produced by the sutures and the local tissue inflammation and edema caused by suturing the mesh is seen to cause more postoperative pain in the polypropylene group [13]. The gripping fixation of the self-gripping mesh provides fixation to the whole of the mesh to the posterior wall. No patients experienced chronic pain at three months post-surgery.

Age distribution

The mean age of patients in the control group was 48 years, compared to 55 years in the study group. A similar study was conducted by Del Papa M et al. [14]. Contrary to the referenced study, the mean age of the study group in our research was higher, yet the complications were fewer. This shows that age is an irrelevant criterion to be compared in compliance with our study.

Side distribution

In our study, we observed that out of a total of 20 patients (40%) with left inguinal hernia, 11 were in the control group and nine were in the study group. Conversely, of the 30 patients (60%) with right inguinal hernia, 14 were in the control group, and 16 were in the study group. This was similar to the study done by Kaya A et al. [15], where right-sided inguinal hernia was more predominant than left inguinal hernia. However, the modality of the surgery did not change, and there was no bias in terms of side selection by the operative surgeon.

Duration of surgery

Being the most important criteria for the prevention of complications of inguinal hernia, it is mandatory to avoid any unnecessary delay in inguinal hernioplasty. In our study, the duration of surgery was less than an hour in 13 patients in the study group and more than one hour in 22 patients in the control group, which was statistically significant. This correlates to the two similar studies conducted by Batabyal P et al. [16] and Del Papa M et al., which also show lesser operative time with self-gripping mesh compared with polypropylene mesh.

Postoperative pain

Understanding the surgical anatomy of the innervation in and around the inguinal canal assists in accurately placing and suturing the mesh. More patients in the control group than in the study group had a pain score of over four. This observation was consistent on all examined post-operative days and aligns with findings from Poobalan AS et al. [17]. A study by Wang Y and Zhang X [18] also reported less post-operative pain with the self-fixing mesh in the early post-operative days. In our study, three months post-surgery, all patients in both the control and study groups had pain scores under four.

Seroma

Seroma may sometimes be mistaken for recurrent inguinal hernia by the patient, leading to unnecessary intervention. In our study, one patient in the control group developed a post-operative seroma, which is consistent with the study by Kazzam ME and Ng P [19], where there were no significant differences in seroma rates between the study and control groups. Seroma should be viewed as a potential post-operative adverse event rather than a complication. It's ideal to avoid aspiration, as it can introduce an infection.

Wound infection

In our study, there was no incidence of wound infection. This contrasts with the study done by Sanders DL et al. [20], where the incidence of wound infection was 2.01% in the control group and 7.19% in the study group. A shorter duration of surgery leads to less tissue exposure, thereby reducing the chances of wound infection.

Foreign body sensation

We also observed that there was no incidence of foreign body sensation, with similar results in line with studies done by Rai S et al. and Yilmaz A et al.

Recurrence

The patients were followed for a period of three months with the primary motive of assessing the duration of surgery. They were also followed up at six months and one year for recurrence. A shorter follow-up period was adopted in view of the early adoption of this technique that will benefit the patients. In our study, recurrence was checked at three months, six months, and at the end of one year. No recurrence of the hernia was observed in patients from both groups. Recurrence post-open Lichtenstein hernioplasty is rare with any type of mesh, whether polypropylene, vypro (vicryl-polyglactin, polypropylene), or self-gripping mesh. Moreover, recurrence at three months is uncommon [21]. It typically arises due to poor patient compliance with post-surgery advice aimed at not raising the intraabdominal pressure (e.g., avoiding constipation, straining during defecation, weight lifting, and chronic cough). Studies by Sanders DL et al. and Yilmaz A et al. also reported no recurrences in both groups. The monitoring period for recurrence was kept short to incorporate this technique into our practice as soon as possible, benefiting the patients. Overall, the results of this study show the advantages of self-gripping mesh. The duration of surgery is significantly shorter. This is because the time taken to fix the mesh is less than one minute on average in the self-gripping mesh. In fixation with polypropylene, the mesh needs suturing with polypropylene with the inguinal ligament, pubic tubercle, conjoint tendon, and fishtailing, which takes time. The short time necessary for mesh fixation reduces the time of mesh exposure and the chances of mesh infection and sepsis [22].

Limitations

This study is a single-center study with a small sample size. To further validate the results, it could be expanded to a multicentric study or include a larger population, or both. Improper mesh placement is challenging since repositioning the mesh damages its gripping surface. This necessitates proper training in mesh handling and ensuring adequate exposure during its placement. Existing collagen disorders in individuals might cause mesh migration. As part of the study's continuation, a long-term follow-up and a correlation between duration and pain scores could be included.

## Conclusions

We conclude that self-gripping mesh is superior to polypropylene mesh in surgery of inguinal hernia in terms of shorter duration of surgery. The shorter operating time, thereby, can be utilized in high-risk individuals who cannot or might have a high risk of being under general anesthesia. This also proves that patients could be taken under local anesthesia with post-operative analgesia, circumventing one spinal prick or intubation. Furthermore, there is a reduction in pain in the immediate postoperative period, although this difference is not statistically significant. Early recovery due to reduced duration and pain leads to a decreased analgesia dosage, making this approach favorable for individuals with potential hepatotoxic or nephrotoxic reactions. Minimal tissue damage reduces the risk of mesh infection. As a result, we have observed improvements in the overall quality of life and a quicker return to normal activities in the study group. Lack of mesh rejection or absence of foreign body sensation despite the added self-gripping morphology of the mesh compared to the conventional one augments the implementation of this technique. There is no considerable difference in both groups regarding seroma formation, wound infection, foreign body sensation, and recurrence. However, a continuation of this study to a larger population group is required to get statistical significance in other criteria and further strengthen these results.

## Appendices

Appendix 1

**Proforma**

**Comparative Study of Self-Gripping Mesh vs Polypropylene Mesh in Lichtenstein’s Open Inguinal Hernioplasty**

NAME:

AGE:

SEX:

OCCUPATION:

ADDRESS:

REGISTRATION NO:

DATA OF ADMISSION:

DATE OF DISCHARGE:

INTRA-OPERATIVE FINDINGS:

TYPE OF MESH USED:

         **POLYPROPYLENE MESH / SELF-GRIPPING MESH**

PAIN SCORE on POD 0 , POD 1 and POD 2 - Less than 4/More than 4

PAIN SCORE AT 3 MONTHS

SEROMA COLLECTION

WOUND INFECTION

FOREIGN BODY SENSATION

RECURRENCE

DURATION OF SURGERY
